# Supplementary material for: Sex Differences in Frailty Factors and Their Capacity to Identify Frailty in Older Adults Living in Long-Term Nursing Homes
Source: Int J Environ Res Public Health. 2022 Dec 21;20(1):54. doi: 10.3390/ijerph20010054 (PMC9819974; doi:10.3390/ijerph20010054)
Supplement: Supplementary file 1 [file ijerph-20-00054-s001.zip › Supplementary File S1_FFP.pdf]

Supplementary File S1. Measures used to assess the Fried Frailty Phenotype (FFP) (6).

	Male	Female
**Unintentional weight loss**	>10 lbs (4.5 kg) or 5% of weight loss in the last year	
**Slow gait speed (4 m)**	Height ≤173 cm: ≥7 s<br>Height >173 cm: ≥6 s	Height ≤159 cm: ≥7 s<br>Height >159 cm: ≥6 s
**Weakness: dominant handgrip strength (kg)**	BMI ≤24.0: ≤29<br>BMI 24.1–26.0: ≤30<br>BMI 26.1–28.0: ≤30<br>BMI >28.0: ≤32	BMI ≤23.0: ≤17<br>BMI 23.1–26.0: ≤17.3<br>BMI 26.1–29.0: ≤18<br>BMI >29.0: ≤21
**Physical activity**	<383 kcal/week (~2.5-h walk)	<270 kcal/week (~2-h walk)
**Exhaustion**	Poor endurance and energy, self-reported from Goldberg Anxiety and Depression Scale	

BMI: body mass index
