# Supplementary material for: Sex Differences in Frailty Factors and Their Capacity to Identify Frailty in Older Adults Living in Long-Term Nursing Homes
Source: Int J Environ Res Public Health. 2022 Dec 21;20(1):54. doi: 10.3390/ijerph20010054 (PMC9819974; doi:10.3390/ijerph20010054)
Supplement: Supplementary file 1 [file ijerph-20-00054-s001.zip › Supplementary File S2_TFI.pdf]

Supplementary File S2. Questions used to assess the Tilburg Frailty Indicator (TFI) (29).

Physical components

1. Do you feel physically healthy?

2. Have you lost a lot of weight recently without wishing to do so? ("a lot" is ≥6 kg during the last six months or ≥3 kg during the last month)

Do you experience problems in your daily life due to:

3. …difficulty in walking?

4. …difficulty maintaining your balance?

5. …poor hearing?

6. …poor vision?

7. …lack of strength in your hands?

8. …physical tiredness?

Psychological components

9. Do you have problems with your memory?

10. Have you felt down during the last month?

11. Have you felt nervous or anxious during the last month?

12. Are you able to cope with your problems well?

Social components

13. Do you live alone?

14. Do you sometimes miss having people around you?

15. Do you receive enough help from other people?
