# Supplementary material for: Sex Differences in Frailty Factors and Their Capacity to Identify Frailty in Older Adults Living in Long-Term Nursing Homes
Source: Int J Environ Res Public Health. 2022 Dec 21;20(1):54. doi: 10.3390/ijerph20010054 (PMC9819974; doi:10.3390/ijerph20010054)
Supplement: Supplementary file 1 [file ijerph-20-00054-s001.zip › Supplementary File S3_scoring details TFI.pdf]

Supplementary File S3. Scoring of the Tilburg Frailty Indicator (TFI) (29).

	**Item**	**Answer Scoring**
Item 1	Good physical health	Yes = 0 No = 1
Item 2	Unexplained weight loss	Yes =1 No = 0
Item 3	Difficulties in walking	Yes = 1 No = 0
Item 4	Difficulties in maintaining balance	Yes = 1 No = 0
Item 5	Poor hearing	Yes = 1 No = 0
Item 6	Poor eyesight	Yes = 1 No = 0
Item 7	Lack of hand strength	Yes = 1 No = 0
Item 8	Physical tiredness	Yes = 1 No = 0
Item 9	Problems with memory	Yes = 1 Sometimes = 0 No = 0
Item 10	Feeling down	Yes = 1 Sometimes = 1 No = 0
Item 11	Feeling nervous or anxious	Yes = 1 Sometimes = 1 No = 0
Item 12	Ability to cope with problems	Yes = 0 No = 1
Item 13	Living alone	Yes = 1 No = 0
Item 14	Lack of social relations	Yes = 1 Sometimes = 1 No = 0
Item 15	Social support	Yes = 0 No = 1
