# Supplementary material for: Sex Differences in Frailty Factors and Their Capacity to Identify Frailty in Older Adults Living in Long-Term Nursing Homes
Source: Int J Environ Res Public Health. 2022 Dec 21;20(1):54. doi: 10.3390/ijerph20010054 (PMC9819974; doi:10.3390/ijerph20010054)
Supplement: Supplementary file 1 [file ijerph-20-00054-s001.zip › Supplementary File S4_Barthel Index.pdf]

Supplementary File S4. Measures for the Barthel Index (27).

FEEDING

0= unable
5= needs help cutting, spreading butter, etc, or requires modified diet
10= independent

BATHING

0=dependent
5= independent (or in shower)

GROOMING

0= needs to help with personal care
5= independent face/hair/teeth/shaving (implements provided)

DRESSING

0= dependent
5= needs help but can do about half unaided
10= independent (including buttons, zips, laces, etc.)

BOWELS

0= incontinent (or needs to be given enemas)
5= occasional accident
10= continent

BLADDER

0= incontinent, or catheterized and unable to manage alone
5= occasional accident
10= continent

TOILET USE

0= dependent
5= needs some help, but can do something alone
10= independent (on an off, dressing, wiping)

TRANSFERS (BED TO CHAIR AND BACK)

0= unable, no sitting balance
5= major help (one or two people, physical), can sit
10= minor help (verbal or physical)
15= independent

MOBILITY (ON LEVEL SURFACES)

0= immobile or < 50 yards
5= wheelchair independent, including corners, > 50 yards
10= walks with help of one person (verbal or physical) > 50 yards
15= independent (but may use any aid; for example, stick) > 50 yards

STAIRS

0= unable
5= needs help (verbal, physical, carrying aid)
10= independent
