# Supplementary material for: Sex Differences in Frailty Factors and Their Capacity to Identify Frailty in Older Adults Living in Long-Term Nursing Homes
Source: Int J Environ Res Public Health. 2022 Dec 21;20(1):54. doi: 10.3390/ijerph20010054 (PMC9819974; doi:10.3390/ijerph20010054)
Supplement: Supplementary file 1 [file ijerph-20-00054-s001.zip › Supplementary File S6_Goldberg.pdf]

Supplementary File S6. Questions included in the Goldberg Anxiety and Depression scales (33).

Anxiety Scale

(*Score one point for each "yes"*)

1. Have you felt keyed up, on edge?
2. Have you been worrying a lot?
3. Have you been irritable?
4. Have you had difficulty relaxing?

(If "yes" to two of the above, go on to ask:)

5. Have you been sleeping poorly?
6. Have you had headaches or neck aches?
7. Have you had any of the following: trembling, tingling, dizzy spells, sweating, diarrhoea? (vegetative symptoms)
8. Have you been worried about your health?
9. Have you had difficulty falling asleep?

Depression Scale

(*Score one point for each "yes"*)

1. Have you had low energy?
2. Have you had loss of interests?
3. Have you lost confidence in yourself?
4. Have you felt hopeless?

(If "yes" to ANY question, go on to ask:)

5. Have you had difficulty concentrating?
6. Have you lost weight (due to poor appetite)?
7. Have you been waking up early?
8. Have you felt slowed up?
9. Have you tended to feel worse in the mornings?

Interpretation:

Add Anxiety score, add Depression score. Patients with Anxiety scores of five or Depression scores of two have a 50% chance of having a clinically important disturbance; above these scores, the probabilty rises sharply.
