# Supplementary material for: Sex Differences in Frailty Factors and Their Capacity to Identify Frailty in Older Adults Living in Long-Term Nursing Homes
Source: Int J Environ Res Public Health. 2022 Dec 21;20(1):54. doi: 10.3390/ijerph20010054 (PMC9819974; doi:10.3390/ijerph20010054)
Supplement: Supplementary file 1 [file ijerph-20-00054-s001.zip › Supplementary File S7_QoLAD.pdf]

Supplementary File S7. Measures for the Quality of Life in Alzheimer's Disease (QoLAD) scale (34).

Quality of Life in Alzheimer's Disease cont'd

QOL-AD

UWMC/ADPR/QOL

Aging and Dementia: Quality of Life in AD

Quality of Life: AD

(Participant Version)

Score (for clinician's use only)

ID Number ☐☐☐☐☐☐ | Assessment Number ☐☐ | Interview Date ☐☐ ☐☐ ☐☐ Month Day Year

Instructions: Interviewer administer according to standard instructions. Circle your responses.

					Score
1. Physical health	Poor	Fair	Good	Excellent	
2 Energy	Poor	Fair	Good	Excellent	
3. Mood	Poor	Fair	Good	Excellent	
4. Living situation	Poor	Fair	Good	Excellent	
5. Memory	Poor	Fair	Good	Excellent	
6. Family	Poor	Fair	Good	Excellent	
7. Marriage	Poor	Fair	Good	Excellent	
8. Friends	Poor	Fair	Good	Excellent	
9. Self as a whole	Poor	Fair	Good	Excellent	
10. Ability to do chores around the house	Poor	Fair	Good	Excellent	
11. Ability to do things for fun	Poor	Fair	Good	Excellent	
12. Money	Poor	Fair	Good	Excellent	
13. Life as a whole	Poor	Fair	Good	Excellent	

Comments: ______________________________

____

Total
