# Supplementary figures and images for: Sex Differences in Frailty Factors and Their Capacity to Identify Frailty in Older Adults Living in Long-Term Nursing Homes
Source: Int J Environ Res Public Health. 2022 Dec 21;20(1):54. doi: 10.3390/ijerph20010054 (PMC9819974; doi:10.3390/ijerph20010054)

Supplementary File S5. Short Physical Performance Battery (SPPB) flowchart (66).

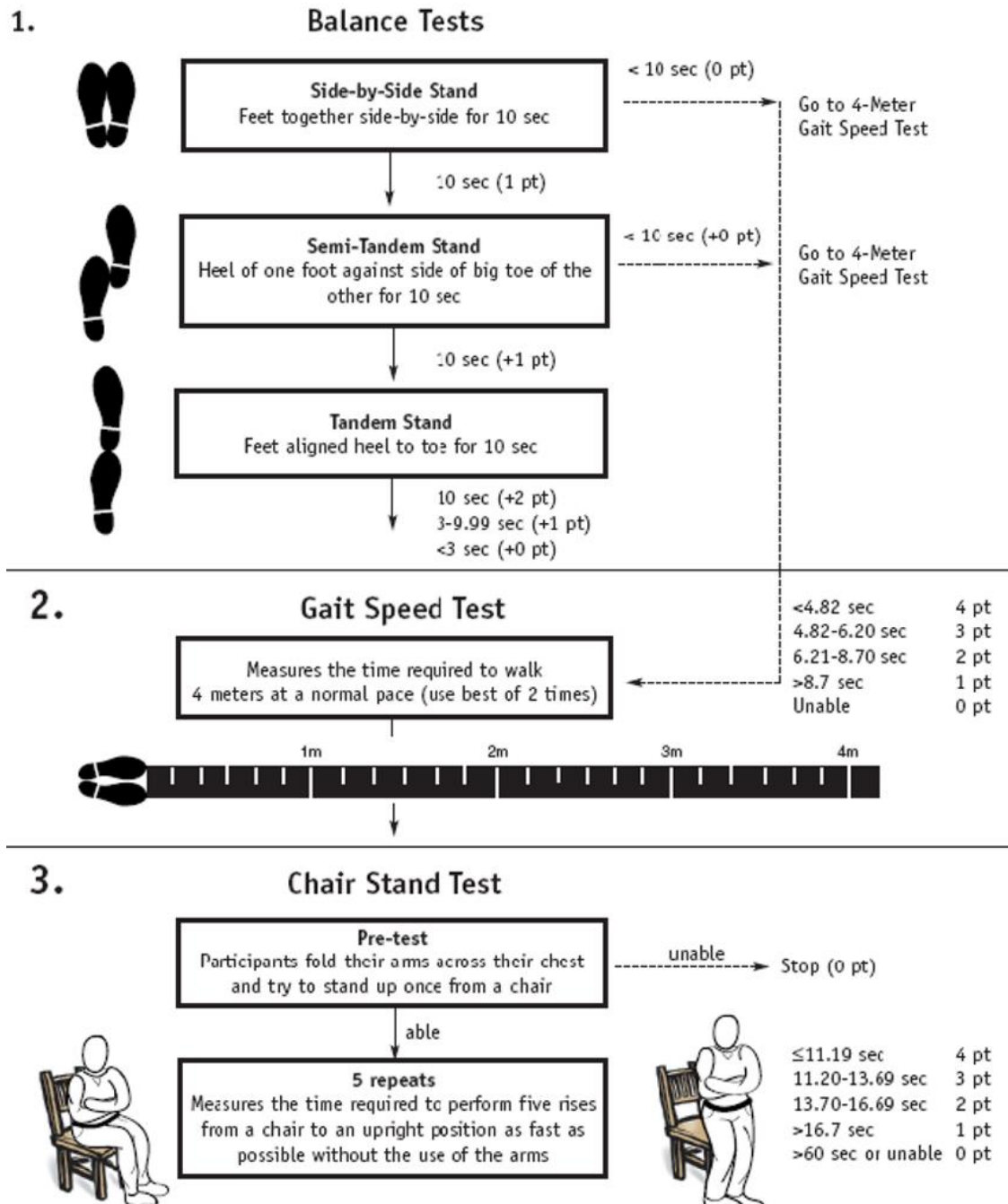

Supplement: Supplementary file 1 [file ijerph-20-00054-s001.zip › Supplementary File S5_SPPB.pdf]
